# Progression from gestational diabetes to type 2 diabetes in one region of Scotland: an observational follow-up study

**DOI:** 10.1186/s12884-015-0457-8

**Published:** 2015-02-03

**Authors:** Claire E Eades, Maggie Styles, Graham P Leese, Helen Cheyne, Josie MM Evans

**Affiliations:** School of Health Sciences, University of Stirling, Stirling, FK9 4LA UK; Department of Diabetes and Endocrinolog, Ninewells Hospital and Medical School, DD1 9SY Dundee, UK; Nursing, Midwifery and Allied Health Professions Research Unit, Unit 13 Scion House, Stirling University Innovation Park, Stirling, FK9 4NF UK

**Keywords:** Gestational diabetes, Type 2 diabetes, Follow-up, United Kingdom

## Abstract

**Background:**

The aim of this study was to investigate long-term risk of type 2 diabetes (T2D) following a diagnosis of gestational diabetes and to identify factors that were associated with increased risk of T2D.

**Methods:**

An observational cohort design was used, following up all women diagnosed with gestational diabetes mellitus (GDM) attending a Diabetes Antenatal Clinic in the Dundee and Angus region of Scotland between 1994 and 2004 for a subsequent diagnosis of T2D, as recorded on SCI-DC (a comprehensive diabetes clinical information system).

**Results:**

There were 164 women in the study who were followed up until 2012. One quarter developed T2D after a pregnancy with GDM in a mean time period of around eight years. Factors associated with a higher risk of developing T2D after GDM were increased weight during pregnancy, use of insulin during pregnancy, higher glycated haemoglobin (HbA1c) levels at diagnosis of GDM, and fasting blood glucose.

**Conclusions:**

These findings suggest there is a viable time window to prevent progression from GDM to T2D and highlights those women who are at the greatest risk and should therefore be prioritised for preventative intervention.

## Background

Gestational diabetes mellitus (GDM) is defined as glucose intolerance that begins or is first detected during pregnancy. GDM can have health consequences for the mother and baby both in the short and longer term. Although normal glucose regulation usually returns shortly after delivery, women diagnosed with GDM have at least a seven fold increased risk of developing Type 2 diabetes (T2D) in the future [1]. In Europe, GDM affects between 2-6% of pregnancies but research has shown that the incidence of GDM has been rising [2,3].

T2D is a growing public health concern associated with a number of serious health complications that reduce both the life-expectancy and quality of life of sufferers [4,5]. There is good evidence to suggest that lifestyle interventions targeted at those at high risk of T2D, such as those with pre-diabetes, can prevent or at least delay the onset of T2D [6]. A diagnosis of GDM therefore represents a window of opportunity for preventative intervention. However, there has been little research on interventions designed specifically for women with GDM, and none in the UK to our knowledge. In order to be able to assess the feasibility and practicality of a lifestyle intervention targeted at women with GDM, it is important to establish the nature of the progression from GDM to T2D in the UK context. A systematic review of studies assessing the association between GDM and T2D did not report any research that had been conducted in the United Kingdom [7]. This study therefore characterises the progression of GDM to T2D in the Dundee and Angus region of Scotland, UK.

## Methods

### Study design and population

This observational study used historical routinely collected health-care data to follow up women diagnosed with GDM. Antenatal care is a universal service accessed by almost all pregnant women in Scotland. Women diagnosed with GDM during routine antenatal care in the Dundee and Angus region (approximate population 250,000) attend the Diabetes Antenatal Clinic at Ninewells Hospital in the city of Dundee. All women in Dundee and Angus were screened with a fasting blood glucose (FBG) or random blood glucose (RBG) at 28 weeks gestation. All patients with any abnormal result (RBG of >5.5 mmol/l^−1^ two or more hours after food or >7.0mmo/l^−1^ within two hours of food; FBG >5.5 mmol/l^−1^), any glycosuria and all high risk pregnancies underwent a 75 g oral glucose tolerance test (OGTT). All women diagnosed with GDM who had attended this clinic between 1994 and 2004, and who had no previous diagnosis of Type 1 or Type 2 diabetes were included in this study. Women diagnosed with GDM in the first trimester of pregnancy were excluded as these women were likely to have had undiagnosed pregestational diabetes [8]. GDM was diagnosed on the basis of clinical guidance in use at the time of the study which suggested an FBG of greater than 5.5 mmol/l^−1^or a blood glucose reading two hours (2 h BG) after an OGTT of greater than 9 mmol/l^−1^.

Data were extracted from paper based case records held at Ninewells Hospital containing clinical and personal data for all women who had attended the diabetes antenatal clinic between 1994 and 2004. These records included the following forms: a booking form which was completed at the first visit to the clinic after a diagnosis of GDM; follow up forms for each further visit to the clinic and a postnatal form containing information from a postnatal check-up. The information extracted from these forms included the mother’s date of birth, family history of diabetes, history of GDM in a previous pregnancy, parity, birth weight of previous babies, week of gestation, OGTT fasting and 2 hour blood glucose levels at booking and postnatal (where recorded), mother’s weight, Hba1C and treatment during pregnancy. Week of gestation, mother’s weight and HbA1c were extracted from the booking, follow up and postnatal forms where recorded.

Data extracted from the paper based records were anonymised and linked to SCI-DC, a validated diabetes clinical information system [9], by the Health Informatics Centre at the University of Dundee (HIC). Patients were followed up for a diagnosis of T2D using the Scottish Care Information – Diabetes Collaboration (SCI-DC) system which holds complete information on patients diagnosed with T2D in Scotland up to March 2012. Women who died or migrated out of the health board during the follow up were not excluded from the study but the date of death/migration was used as their study end date in the analysis.

Patients with T2D are defined as those who are diagnosed with diabetes over the age of 35 years, or younger patients for whom there is no immediate requirement for insulin. World Health Organisation (WHO) criteria were used to diagnose T2D but the precise glucose levels used depended upon the criteria in use at the time of diagnosis. The majority of women included in the study (97%) were diagnosed using the WHO criteria published in 1999 [10] which defines T2D on the basis of a fasting plasma venous sample of 7.0 mmol/l^−1^ or higher and a 2 hour post OGTT value of 11.1 mmol/l^−1^. The remainder were diagnosed using the WHO 1985 criteria [11] which had a higher value for fasting venous plasma of 7.8 mmol/l^−1^ or higher but the same 2 hour value. The data were also linked to a portion of the ISD SMR02 dataset which provided demographic information not available from paper based records such as deprivation category (from the Scottish Index of Multiple Deprivation [12] and body mass index (BMI). The SIMD deprivation category is a postcode measure derived from multiple aspects of deprivation including employment, income, health, education, access to services, crime and housing.

### Analysis

In survival analyses, women were followed up from the date of diagnosis of gestational diabetes. Women who had more than one pregnancy during the study period were followed up from the earliest date of diagnosis of gestational diabetes. The relationships between potential risk factors and development of T2D were assessed by univariate and multivariate Cox regression, from which hazard ratios (HRs) and 95% confidence intervals (CIs) were calculated. Deprivation category, age, history of GDM, family history of diabetes, use of insulin during pregnancy, average weekly weight gain and weight, trimester, HbA1c, FBG, 2 h BG at diagnosis of GDM were entered as independent variables, with diagnosis of T2D as the dependent variable. Statistical analyses were carried out using SPSS for Windows version 21. Ethical approval was obtained from the School of Nursing, Midwifery and Health at the University of Stirling. The Tayside Committee for Medical Research Ethics has granted approval for studies using routinely collected, anonymised health data and this study falls under this approval.

## Results

### Characteristics of population

Data were extracted from the records for 285 women, of which 164 women met the criteria for GDM and had no previous diagnosis of Type 1 or Type 2 diabetes, and were therefore included in the study. Of the remainder, 75 women had Type 1 Diabetes, 12 had Type 2 Diabetes, 2 were diagnosed with GDM in the first trimester and 1 had maturity onset diabetes of the young. A further 21 women were classified as borderline GDM as their blood glucose results were high but did not meet the criteria for GDM. Ten women with GDM were excluded due to having missing data or a previous diagnosis of Type 1 or Type 2 diabetes.

At the time of diagnosis of GDM, women ranged in age from 16 to 43 with a mean age of 30. Table 1 shows further characteristics of the population. Women were more commonly from areas of higher deprivation than lower deprivation. A positive family history of diabetes was noted for around a third of women and the majority were having their first or second child. BMI data were not recorded for the majority of women in this study.

**Table 1:** **Characteristics of the population**

**Deprivation category**	**n (%)**
5 (Lowest Deprivation)	17 (10.4)
4	39 (23.8)
3	21 (12.8)
2	35 (21.3)
1 (Highest Deprivation)	44 (26.8)
Data Missing	8 (4.9)
**Previous live births**	**n (%)**
0	57 (34.8)
1	54 (32.9)
2	28 (17)
3 or more	19 (11.6)
Data Missing	6 (3.7)
**Mother’s weight (kg)**	**n (%)**
Up to 76.8	46 (28)
76.8-92.5	49 (29.9)
Over 92.5	53 (32.3)
Data Missing	16 (9.8)

### Progression to type 2 diabetes

**Figure 1:**
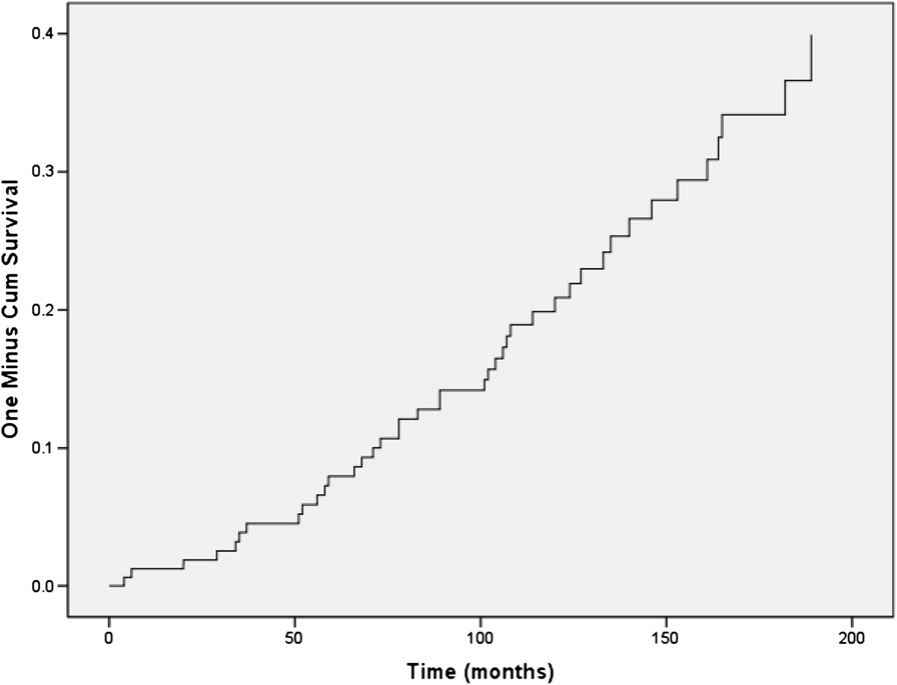
**Cumulative incidence of T2D after diagnosis of GDM.**

Forty one women (25%) developed T2D during follow-up. The time between diagnosis of GDM and T2D ranged from 4 months to nearly 16 years, with a mean time of 93 months (SD = 48.2) or nearly 8 years. Of these women only 3 (7.3%) went on to develop T2D in the two years after their diagnosis of GDM and a further 4 (9.8%) developed T2D two to four years after their diagnosis of GDM. Figure 1 shows a relatively steady rate of T2D incidence after diagnosis of GDM over the study period. Table 2 shows the results of both univariate and multivariate Cox survival analysis. Greater weight during pregnancy, insulin use during pregnancy, higher HbA1c levels and FBG were associated with highly elevated risks of progression to T2D in univariate and multivariate analyses. Although 2 h BG and were also associated with an increased risk univariately, this association was no longer statistically significant after adjusting for other variables. While there were no statistically significant associations for increasing age, family history of T2D or previous history of GDM, the hazard ratios were elevated. There was no evidence for an association with deprivation or average weekly weight gain.

**Table 2:** **Hazard ratio of developing T2D in patients with GDM according to previous history of GDM, family history of diabetes, deprivation category; insulin use and average weekly weight gain during pregnancy; and weight, age, trimester, HbA1c, fasting and 2 hour blood glucose at diagnosis of GDM**

	**Univariate**				**Multivariate**	
	**No. (%) progressing to T2D**	**Mean time to progress (months)**	**Hazard ratio (95% CI)**	**p value**	**Hazard ratio (95% CI)**	**p value**
**Whole sample (n=164)**	41 (25)	93				
**Deprivation category (no. in each group)**						
5 least deprived (17)	2 (12)	21	1.00		1.00	
4 (39)	8 (21)	78	1.44 (0.31-6.76)	0.647	0.68 (0.12-3.95)	0.671
3 (21)	6 (29)	94	1.86 (0.38-9.20)	0.448	0.71 (0.11-4.61)	0.717
2 (35)	13 (37)	115	2.81 (0.63-12.45)	0.174	1.92 (0.37-10)	0.438
1 most deprived (44)	11 (25)	82	1.76 (0.39-8.89)	0.461	0.76 (0.15-3.93)	0.742
Data missing (8)	1 (13)	106	0.81 (0.07-8.89)	0.860	0.9 (0.07-11.96)	0.936
**Age (no.)**						
25 and under (32)	6 (19)	99	1.00		1.00	
26 to 34 (84)	19 (23)	93	1.28 (0.51-3.19)	0.604	1.35(0.48-3.79)	0.570
35 and over (48)	16 (33)	86	1.90 (0.74-4.87)	0.179	2.38 (0.82-6.95)	0.112
**Previous history of GDM (no.)**						
Yes (11)	4 (36)	83	1.7 (0.61-4.77)	0.315	2.83 (0.62-12.87)	0.179
No/Data missing (153)	37 (24)	91	1.00		1.00	
**Family history of diabetes (no.)**						
Yes (59)	17 (29)	89	0.80 (0.43-1.49)	0.485	1.42 (0.64-3.15)	0.385
No/Data missing (105)	24 (23)	93	1.00		1.00	
**Weight (no.)**						
Up to 76.8 kg (46)	3 (7)	70	1.00		1.00	
76.8 to 92.5kg (49)	15 (31)	88	5.19 (1.5-17.93)	0.009	4.98 (1.23-20.18)	0.024
Over 92.5kg (53)	20 (38)	89	6.49 (1.93-21.86)	0.003	5.22 (1.38-19.73)	0.015
Data missing (16)	3 (19)	150	3.05 (0.62-15.12)	0.172	3.5 (0.53-23.34)	0.196
**Trimester at diagnosis (no.)**						
2^nd^ trimester (16)	5 (31)	96	1.21 (0.47-3.07)	0.696	1.05 (0.32-3.45)	0.942
3^rd^ trimester (147)	36 (24)	90	1.00		1.00	
**HbA1c in mmol/mol (no.)**						
33.3 and under (19)	3 (16)	150	1.00		1.00	
33.3 to 42.1 (22)	5 (23)	82	1.42 (0.34-5.95)	0.630	1.59 (0.29-8.84)	0.597
42.1 plus (18)	8 (44)	57	4.41 (1.17-16.69)	0.029	5.34 (0.98-29)	0.052
Data missing (105)	25 (24)	98	1.66 (0.5-5.5)	0.407	1.9 (0.45-7.94)	0.381
**Fasting blood glucose in mmol/l (no.)**						
Under 5.1 (52)	6 (12)	102	1.00		1.00	
5.1 to 7.0 (72)	20 (28)	93	2.62 (1.05-6.53)	0.038	1.66 (0.52-5.24)	0.392
Over 7.0 (17)	6 (35)	60	6.87 (2.2-21.44)	0.001	3.94 (0.92-16.91)	0.065
Data missing (23)	9 (39)	99	3.87 (1.38-10.89)	0.010	35.29 (2.18-570.68)	0.012
**2 hour post load blood glucose in mmol/l (no.)**						
Under 8.5 (39)	7 (18)	83	1.00		1.00	
8.5-11.1 (67)	14 (21)	96	1.15 (0.46-2.85)	0.762	1.54 (0.54-4.38)	0.417
Over 11.1 (36)	12 (33)	84	2.58 (1.01-6.56)	0.047	2.37 (0.76-7.4)	0.139
Data missing (22)	8 (36)	99	2.13 (0.77-5.88)	0.144	0.1 (0.01-1.56)	0.101
**Used Insulin during pregnancy (no.)**						
Yes (51)	20 (39)	88	2.82 (1.52-5.2)	0.001	2.81 (1.35-5.86)	0.006
No (113)	21 (19)	94	1.00		1.00	
**Average weekly weight gain (kg)**						
0.3 and under (61)	16 (26)	82	1.00			
0.31 and above (64)	14 (22)	90	0.71 (0.35-1.47)	0.360	0.61 (0.2-1.45)	0.259
Data Missing (39)	11 (28)	105	1.04 (0.49-2.25)	0.912	1.12 (0.43-2.93)	0.821

## Discussion

To the best of our knowledge this study is the first to investigate progression from GDM to T2D in the UK. We found that around a quarter of women diagnosed with GDM developed T2D with a mean time window between the two diagnoses of 8 years. The vast majority of women who did develop T2D after GDM did so five years or more after their diagnosis of GDM. This time period presents a considerable window of opportunity to deliver an intervention and for women to make necessary changes to their diet and activity levels in order to reduce the risk of progression to T2D. Many people find making lifestyle changes difficult and women who have recently had a baby face additional problems. For example, a lack of time is often cited by women who have had GDM as a barrier to making lifestyle changes [13]. Our findings suggest that the window of opportunity may be large enough for the majority of women to allow an intervention to be delayed until the child is slightly older and less dependent. Such a delay may help to address some of the barriers to lifestyle change faced by women with GDM but this argument becomes complex if women are planning to have more children. This issue is further complicated by the fact some women have already made lifestyle changes during pregnancy in an attempt to manage their GDM. With these women it may be best to intervene sooner after pregnancy to ensure these changes are maintained. The timing of lifestyle interventions for women who have had GDM clearly needs further exploration with women, along with the optimal content and means of delivery, if interventions are to be successful.

Women who were at highest risk of developing T2D after GDM were heavier women, those with an HbA1c of over 42.1 mg/dL, those who used insulin during their pregnancy and those with FBG of 7.0 mmol/l and over. These women should arguably be prioritised for intervention. These findings are largely consistent with previous research reported in a systematic review of studies assessing the incidence of T2D after a diagnosis of GDM [7].

Higher FBG levels and HbA1c were associated with higher risk univariately, but this increased risk was only marginally significant in the multivariate analysis. However, we identified an increased risk of four fold for women who had an FBG of 7.0 and over five fold for women with an HbA1c of over 42.1 mg/dL. Given the small sample size and wide confidence intervals in this study, these marginally significant risks cannot be discounted. It is difficult to compare our finding for FBG with previous research that has generally looked at FBG as a continuous variable; thus particular thresholds of FBG for increased risk of T2D have been difficult to pinpoint. Studies that did use categories for FBG reported varying findings. One study found an 11 fold risk in women who had an FBG of 5.9 or over compared to those with lower FBG values [14]. Two other studies reported that women who went on to develop T2D had a mean FBG of closer to 8.0 [15,16].

The systematic review of studies assessing the incidence of T2D after a diagnosis of GDM [7] reported mixed findings for the association between BMI and future T2D risk. There were insufficient data for BMI in the present study to include it in the survival analysis. However, weight was found to be significantly associated with increased risk of T2D in the multivariate analysis, with other factors such as trimester controlled for. Although weight is typically regarded as an unreliable measure of obesity and disease risk as it does not take into account height, our study does suggest that it may be a useful indicator of future risk of T2D in women with GDM.

We did not find statistically significant associations between increasing age, history of GDM in a previous pregnancy or family history of diabetes and future risk of T2D. Although the hazard ratio estimates were elevated, particularly for previous history of GDM, and therefore increased risks cannot be discounted, the sample size in our study was relatively small and confidence intervals were wide. Previous research reports mixed results for these risk factors; therefore larger studies are required to verify the results.

Despite being a small study, the diagnosis of GDM in our sample of 164 women was validated for each one and we are confident in the high quality of our data. Detailed information was collected from paper records using a pre-defined data collection tool. The subsequent T2D diagnoses were made using a diabetes clinical information system that has been extensively used in health care research and is known to be accurate. However, with around 2,600 births per year in Dundee and Angus, it is clear that we did not identify all cases of GDM during the study period. We would have expected to identify between 500 and 600 women over the period of the study using a conservative rate of 2% of pregnancies affected by GDM. On the other hand, we know that the women that we did include definitely had GDM, even if they represent a sample only. Reasons for the low number of women identified with GDM might include the non-universal screening of women for GDM, ‘lost’ paper-based records, women attending other diabetes antenatal clinics in the region or women treated solely in primary care or general antenatal clinics. It is also likely that a proportion of women who had GDM went undiagnosed due to lower awareness of the condition in the past. Another limitation of this study was the high level of missing data in the paper records for several of the variables of interest which limited our ability to investigate them in depth. Despite these limitations, this is the first study of its kind to be carried out in the UK. The region in which the study was carried out is broadly representative of the total population of Scotland and the results are more generalizable to the UK than similar studies in Europe and the United States.

## Conclusions

In summary, this study clearly shows how a diagnosis of GDM can have an adverse impact on health that extends long after the pregnancy. This study highlights those women with GDM who are at the greatest risk of progressing from GDM to T2D and should therefore be prioritised for preventative intervention and suggests there is a viable time window to prevent progression from GDM to T2D in the majority of women. While a diagnosis of GDM presents an ideal opportunity for an intervention to reduce the growing burden of T2D, identifying the most effective way and optimal time to help women who are at a particularly busy period of their lives to engage in lifestyle change remains a challenge that needs further exploration.

## Abbreviations

2 h BG: 2 hour blood glucose
BMI: Body mass index
FBG: Fasting blood glucose
GDM: Gestational diabetes mellitus
Hba1c: Glycated haemoglobin
OGTT: Oral glucose tolerance test
SCI-DC: Scottish care information – diabetes collaboration
SIMD: Scottish index of multiple deprivation
T2D: Type 2 diabetes
WHO: World Health Organisation
RBG: Random blood glucose
